# A framework for sustainable capacity-building for collaborative North–South translational health research and training in a resource-constrained setting

**DOI:** 10.1186/s12961-023-00972-0

**Published:** 2023-03-27

**Authors:** Charles C. Maponga, Alison T. Mhazo, Gene D. Morse

**Affiliations:** 1 Department of Pharmacy and Pharmaceutical Sciences, University of Zimbabwe, Faculty of Medicine and Health Sciences, Harare, Zimbabwe; 2 Ministry of Health, Community Health Sciences Unit, Private Bag 65, Area 3, Lilongwe, Malawi; 3 Center for Integrated Global Biomedical Sciences, University at Buffalo, State University of New York, Buffalo, NY USA

**Keywords:** Capacity-building, Policy entrepreneurship, Mnemonic acronym, North–South research collaboration

## Abstract

**Introduction:**

Success with highly active antiretroviral therapy (ART) for the human immunodeficiency virus (HIV) in developing countries has been attributed to collaborative North–South resource-sharing and capacity-building. Academic research and training programmes have contributed towards policy entrepreneurship in a manner that influenced capacity-building within health systems. However, the documented capacity-building frameworks rarely elucidate how such programmes can be designed and implemented efficiently and sustainably.

**Method:**

We implemented the University of Zimbabwe (UZ)–State University of New York at Buffalo (UB) collaborative HIV clinical pharmacology capacity-building programme in Zimbabwe in 1998. We intuitively operationalized the programme around a mnemonic acronym, “RSTUVW”, which spells out a supportive framework consisting of “room (space), skills, tools (equipment)”, underpinned by a set of core values, “understanding, voice (clout) and will”. Subsequent to our two decades of successful collaborative experience, we tested the general validity and applicability of the framework within a prospective programme aimed at expanding the role of health professionals.

**Results and conclusion:**

Based on this collaborative North–South research and training capacity-building programme which has been positively validated in Zimbabwe, we propose this novel mnemonic acronym-based framework as an extra tool to guide sustainable capacity-building through collaborative North–South implementation research. Its extended use could also include assessment and evaluation of health systems within resource-constrained settings.

## Introduction

Health sector capacity-building is the development of knowledge, skills, commitment, structures, systems and leadership to promote public health. It involves the advancement of knowledge and skills among practitioners, the expansion of support and infrastructure and the development of partnerships [1]. Building research capacity in health services has been recognized internationally as important to produce a sound evidence base for decision-making in policy and practice [2]. WHO, the Council on Health Research for Development (COHRED), the Global Forum on Health Research (GFHR) and other agencies concerned with international health have consistently emphasized that a primary function of sustainable knowledge systems is to create and continuously improve the human and physical resources for health research [3]. Owing to its disproportionate epidemiological and socioeconomic impact in developing countries, HIV/AIDS has attracted considerable research attention since the 1980s, with a predominant focus on public health, epidemiology and drug therapy [4]. In the wake of an unprecedented upsurge in research interest, the HIV/AIDS epidemic also revealed glaring research capacity gaps between developing and developed countries. As a result, an archetypical feature of HIV/AIDS research has been the establishment of North–South (N–S) research collaborations whereby researchers from a developed country (North) and a developing country (South) agree to conduct research jointly [5].

At its core, N–S collaboration is a mechanism to channel resources to support scientific and technological activities in resource-constrained countries. The collaboration may involve two institutions (universities, research organizations, government agencies, etc.) conducting joint research in a single country or in multiple institutions in several countries [6]. Approaches to N–S collaboration are correlated with different historical backgrounds and political climates for foreign scientific and technical assistance [5]. Countries such as France, the United Kingdom, the Netherlands, Belgium, Germany, Portugal and Spain having a long history of rendering scientific and technical assistance to developing countries, and starting from the 1950s and even more during the 1960s, the involvement of the United States of America became a significant and dominant force [5].

### Policy entrepreneurship and N–S collaborations

Like any other initiatives involving change, the success or failure of N–S collaborations hinges on institutional entrepreneurship—an organizational science and management principle that explains how change can be initiated and sustained in an organization [7]. Institutional entrepreneurship is driven by individual actors—known as institutional entrepreneurs—who rely on existing capacities within their context, their motivation for change, ability to frame issues and how much they can draw on or exert power and influence [7]. This is analogous to John Kingdon’s conceptualization of policy entrepreneurs—individuals who take advantage of favourable moments to strategically devote their resources (energy, commitment and intellect) to drive policy change [8–10]. The role of policy entrepreneurs has been identified as a key component in reforming health policies in Africa [11], and within the context of openness to policy development, local researchers have acted as policy entrepreneurs by bringing attention to priority health issues and translating research evidence into policy [12].

In the Zimbabwean context, individual institutional and policy entrepreneurs have been credited for the continuity of the University of Zimbabwe (UZ)-State University of New York at Buffalo (UB) collaborative programme since its inception in 1998 [13]. Thus, academic institutions, complemented by their respective international collaborators, and favoured by credibility for neutrality and capacity to advance knowledge, have long been recognized as strategic in driving policy entrepreneurship to solve pressing societal problems in Africa [14–19]. This also includes promoting contemporary development paradigms such as the Sustainable Development Goals (SDGs) [15, 17]. Whilst the above account has presented policy entrepreneurs in academic institutions as a source of policy change, it is also important to cast their role as policy catalysts through the lens of collaborative entrepreneurship with nonacademic institutions. For example, after global policy entrepreneurs such as WHO have conceived policy norms, academic institutions act as policy diffusion entrepreneurs by conducting research that informs key aspects of implementation in the real world, such as feasibility and acceptability in a given context [20].

In Zimbabwe, academic scientists have been at the forefront of conducting in-country studies on the HIV/AIDS treatment regimens recommended by WHO, which has not only supported optimization of pharmacotherapy within local clinical settings [21, 22], but also influenced WHO recommendations at a global scale [23]. The role of academic institutions as part of collaborative entrepreneurship has also been facilitated through the active championship of individual policy entrepreneurs such as the former secretary-general of the United Nations, Kofi Annan, who underscored the “need for a true partnership of developed and developing countries—a partnership that includes science and technology” [24].

### Capacity-building within N–S collaborations

Despite the increase in N–S capacity-building initiatives and their influence on policy in developing countries, a systematic review conducted in 2017 concluded that there was limited use of clearly identified, referenced and outlined theories, models or frameworks to support capacity-building interventions [25]. This paper draws from 20 years of experience in implementing the UZ and UB collaborative clinical pharmacology capacity-building programme [13] to propose a framework that can be systematically used to plan and analyse the effectiveness and sustainability of such initiatives. We provide a brief overview of the implementation of N–S research collaborations and then situate academic institutions as a source of policy entrepreneurship. We use our 20-year implementation experience with the UZ-UB collaboration to propose a framework for sustaining capacity-building initiatives in a resource-constrained setting. We also provide a descriptive overview of the capacity-building initiative, focusing on its historical origins and major features. We retrospectively test the framework of the UZ-UB initiative we implemented in Zimbabwe. Specifically, we show how the elements of the framework could be used as explanatory variables for the continuity of the collaboration and its policy impact. We conclude by prospectively presenting how the framework can be used to strategically frame, introduce and scale up implementation research to achieve best practice in pharmacotherapy.

### The collaborative HIV/AIDS clinical pharmacology capacity-building programme in Zimbabwe

**Fig. 1:**
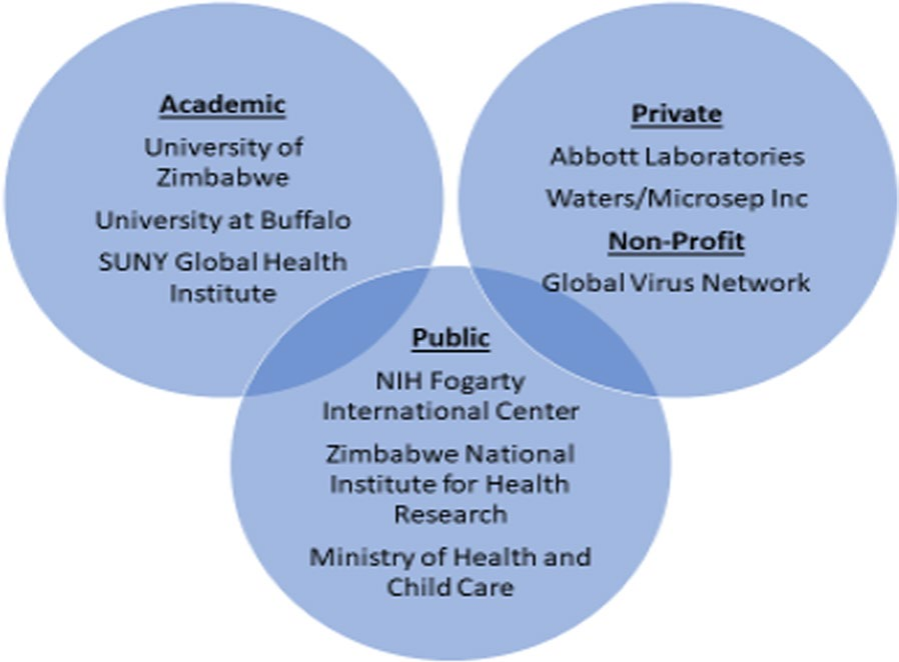
Academic-public–private partnership for HIV pharmacotherapy capacity-building in Zimbabwe

In 1998, UZ and UB developed a collaborative clinical pharmacology capacity-building programme in Zimbabwe to train the next generation of HIV researchers and support the rollout of the national HIV programme [13]. Through funding from the National Institutes of Health/National Institute for Allergy and Infectious Diseases (NIH/NIAID), the pioneering capacity-building initiative involved a mentored programme for a postdoctoral fellowship for the then chairman of the UZ Department of Pharmacy through a UZ-UB collaborative partnership. Figure 1 shows a schematic of the academic, private and public partnerships that shaped the capacity-building collaboration. For the academic sector, UZ and UB, through the State University of New York (SUNY) Global Health Institute, had a formal memorandum of understanding (MOU) that guided the collaboration. The collaboration involved mentored fellowships, student exchange programmes, joint research and collaborative publication authorship. Fellowships spanned various areas of HIV/AIDS pharmacotherapy including pharmacokinetic studies, antiretroviral therapy (ART) optimization and rationalization of herbal therapy in people on ART.

Key success outcomes of the collaboration included (1) strengthening UZ faculty scholarly capacity and retention of human resources, (2) technology and skills transfer to support research and practice, (3) strengthening the HIV clinical pharmacology evidence base for practice and policy, and (4) local partnerships to support international collaboration. Some of the challenges experienced included policy inconsistencies linked to political cycles, and socioeconomic difficulties, as well as the evolving nature of HIV/AIDS as a global public health issue.

The public sector included the NIH Fogarty International Center in the United States and the Zimbabwe National Institute for Health Research within a policy environment supported by the Ministry of Health and Child Care. Key success experiences and outcomes in relation to collaboration with the public sector include swifter and wider dissemination of approved policies. However, lingering policy shifts and sociopolitical and economic challenges were encountered. For-profit, private entities and not-for profit institutions supported the collaboration with funding and infrastructure to facilitate collaborative research. Key success experiences and outcomes in relation to collaboration with the private sector included technology transfer through donation of laboratory equipment and funding for specific projects such as clinical trials. With these private sector collaborations, some challenges were encountered such as limitations in the scope of focus associated with the private sector’s profitability-dependent models.

At the launch of the UZ-UB capacity-building programme in 1998 and during its formative years, the science of HIV/AIDS pharmacotherapy was still developing and the feasibility of rolling out ART in developing countries was plagued with scientific doubt and scepticism [26]. Despite the scepticism, significant progress was made from the mid-2000s. Success has been attributed to collaborative research, training and service activities in a variety of areas including HIV/AIDS clinical pharmacology [27]. Promotion of capacity-building through various organizations such as the Fogarty International Center AIDS International Training and Research Program (AITRP) has been undertaken to sustain such gains [28].

### A framework for designing, implementing and sustaining capacity-building for translational health research collaborations

After a decade of implementing the UZ-UB capacity-building programme, we adopted RSTUVW (room, skills, tools, understanding, voice, will) as a framework to guide the implementation of an AITRP focused on clinical pharmacology, with priorities such as HIV prevention and treatment research, and coinfections that included tuberculosis, malaria and hepatitis. The framework was presented to HIV/AIDS global stakeholders during the 2011 International AIDS Society Conference held in Rome, Italy [29]. We present each element of the framework below in the context of AITRP implementation. The core values (understanding, voice and will) are discussed together due to their close association and complementarity. We intuitively operationalized the programme around a mnemonic acronym, “RSTUVW”, which spells out a supportive framework consisting of “*r*oom (space), *s*kills, *t*ools (equipment)” underpinned by a set of core values, “*u*nderstanding, *v*oice (clout) and *w*ill. We opted to use a mnemonic for three principal reasons. First, mnemonics are potent memory aids that have been used for at least 2500 years and have recently been studied experimentally and used in a variety of disciplines [30, 30–33]. Second, the field of cognitive psychology considers mnemonic techniques as encoding strategies for easy retrieval of new information from memory [30]. Among the most commonly studied techniques are those involving verbal mnemonics, such as using the first letters of a set of words to form an acronym or phrase, or using the words to make up useful statements.

**Fig. 2:**
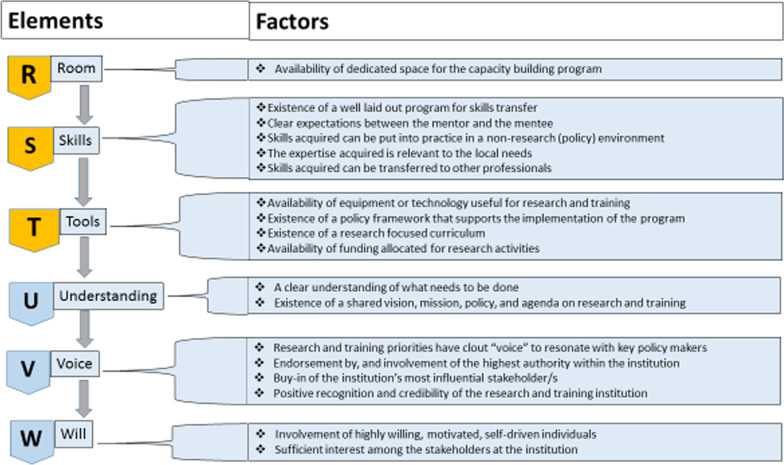
RSTUVW framework for sustainable capacity-building for translational North–South health research collaborations in resource-constrained settings

Some mnemonic techniques take advantage of the benefits of meaningful and organized encoding, and supplement them by setting up an organized retrieval structure in which each retrieval cue is stored with a specific piece of information to be remembered. To be maximally effective, these cues must be memorable and have a good likelihood of reminding the individual of the target information [32]. Third, mnemonics in education and training have been a research-based tool to convert difficult-to-remember concepts into more memorable ones. It works by using easy-to-remember lines such as the sequencing of letters of the alphabet to help students learn a significant amount of information as well as increase long-term retention [30]. The use of mnemonics to simplify complex issues in cognitive theory is akin to the use of metaphors in policy-making. In policy-making, pioneering ideas are often presented as metaphors that can be used by policy-makers to boil down a set of complex policy trade-offs into a few consistent strategies and principles [34]. Thus, some influential theories of policy change are replete with metaphors such as policy streams and policy windows [9], an attribute that has been associated with their cognitive appeal and wider applicability in diverse policy spheres globally [35]. The RSTUVW framework is captured in Fig. 2.

### Elements of the RSTUVW framework

The elements of the RSTUVW framework are described below.

#### Element 1: room

Lack of infrastructure is singled out as one of the most important barriers to carrying out scientific research in Africa [36]. Medical research relies on appropriate infrastructure to house research equipment, facilitate flow of research materials and protect the researcher from the biohazards associated with working in a biomedical research laboratory. Lack of appropriate infrastructure partly explains the low level of research output in Africa, accounting for only 2% of the world’s research output and 1.3% of global publications [37]. We attribute the availability of room (space) for pharmaceutical bioanalysis, UZ-International Pharmacology Specialty Laboratory, pharmacokinetic and pharmacology studies, and support group activities as a key success factor for the UZ-UB capacity-building in Zimbabwe.

#### Element 2: skills

Suboptimal research capacity in African countries is mainly attributable to inadequacies in skills [37]. This is compounded by poor supervision of higher-degree scholars, weak or very limited progression pathways for those in scientific careers, and brain drain [38]. The devastating impact of HIV/AIDS in sub-Saharan Africa and lack of research capacity has highlighted the importance of building local capacity for scientific and health systems research. Within the context of N-S collaborations, it also meant that research had to be relevant and responsive to local needs. The UZ-UB collaboration was conceived to build capacity for research that was relevant to address the local HIV/AIDS pharmacotherapy priorities within the context of fulfilling an urgent unmet medical and societal need. Since its inception, 38 fellows have been trained and mentored across a portfolio of areas to meet differentiated, locally tailored policy priorities spanning paediatric therapy, ethics, economic aspects, health information and medicine safety [20].

The approach involving the training of scholars driven by policy needs had an impact on the fellows’ individual career progression and their policy impact. At an individual level, some alumni of the UZ-UB collaboration have progressed to occupy faculty positions at local academic institutions, while others are leading HIV/AIDS research within service delivery environments. At a policy level, fellows and ex-fellows have been incorporated into strategic technical working groups that oversee the optimization of ART in Zimbabwe. The policy impact has also been amplified by an appreciation of the interface of local sociocultural circumstances (including related indigenous knowledge systems) and HIV pharmacotherapy. For example, an appreciation that supplementation of conventional medicines with herbs is prevalent amongst people infected with HIV led to focused studies that examined the prevalence of the practice in Zimbabwe, its effect on ART metabolism and the potential toxicities associated with ART–herbal interactions [39–41].

#### Element 3: tools

In this framework, tools encompass relevant policy frameworks, funding and equipment. Regarding funding, sub-Saharan Africa accounts for 10% of the global population and only 1.3% of global health research publications, an inverse scenario that has been attributed to lack of funding [42]. Lack of dedicated funding for research therefore heavily undercuts both operational feasibility and human motivation. From 2002 to the present, the UZ-UB collaboration has been guided by policy frameworks in the form of three successive MOUs. The first MOU (2002–2007) and the second MOU (2009–2015) were between the UZ and the UB AIDS Research and Training Program (AITRP) whilst the current MOU (2016–2021) is between UZ and the UB HIV Research and Training Program (HRTP). The successive MOUs between UZ and the AITRP and HRTP were accompanied by grants (Grant Numbers D43TW010313, D43TW007991, D43TW007991 01A2S1 and 2D43TW010313-06) that supported formalized mentoring, joint research projects, joint research publications, participation in conferences and workshops, and opportunities for higher education and scholarly internships. At a research systems strengthening level, the funding also enabled the provision of the other tools, particularly laboratory equipment and reagents, to carry out essential research in areas such as bioequivalence studies and therapeutic drug monitoring [43]

#### Elements 4–6: core values (understanding, voice and will)

Whilst tangible physical space (room), skills and tools are critical for sustaining N–S health research capacity-building programmes, we found core intangible values that drive the successful implementation of such programmes. Fostering of core values is achieved through workshops, to promote a clearer grasp (understanding) of the goals of the project, while seeking the support and endorsement of these goals to the highest authorities with clout (voice) in the university to stimulate greater desire (will) among the various stakeholders.

The UZ-UB collaboration promoted clearer understanding of the goals of the project through formalized mentoring of the research fellows, exchange programmes and the establishment of clear linkages between the skills acquired and policy input. By linking the programme to its strategic role in addressing a pressing public problem (high HIV/AIDS morbidity and mortality) and articulating the vision of the programme (support the provision of life-saving ART), the voice of those who designed the programme resonated with the authorities at UZ and the Ministry of Health and Child Care to secure higher-level endorsement. This is in concert with influential theories that explain the generation of political priority for policy matters [44, 45]. With a well-articulated vision backed by high-level endorsement, the UZ-UB collaboration attracted the will of a broad group of stakeholders involved in shaping health research in Zimbabwe. The interest of wider stakeholders was key in establishing strategic domestic ties. For example, the Medicines Control Authority of Zimbabwe (MCAZ) provided space (room) at one of its premises to relieve space constraints at the UZ-UB collaboration’s traditional site at the UZ Faculty of Medicine and Health Sciences. At a policy level, as stated earlier, the scholarly contribution of the collaboration to policy continues to be valued through participation of fellows in ART technical working groups. We also identified that the generation and fostering of the core values of understanding, voice and will relied on the active participation of policy entrepreneurs—individuals who take advantage of favourable moments to further policy ends by strategically attaching problems to solutions [8, 9].

## Discussion

This paper has presented a novel mnemonic-based capacity-building framework that was informed from a research environment with potential use in wider health system settings. The framework shares common conceptual underpinnings or themes that have been identified in other research capacity-building frameworks, namely supporting individuals in research, collaborations and valuing research excellence [2, 46, 47]. The framework also shares some similarity with frameworks derived from general public policy contexts. Wu et al. developed a conceptual framework for capacity-building in terms of competencies and capabilities where competencies are categorized into three general types of skills essential for policy success—analytical, operational and political—while policy capabilities are assessed at the individual, organizational and system resource levels [48]. We posit that analytical, operational and system resource aspects are closely related to the tangibles in the RSTUVW framework, whilst aspects such as political skills are more inclined towards the intangibles. We also found overlaps with existing conceptualizations on South–South, N–S and North–South–South collaborations which identified the strategic, tactical and operational domains in such collaborations cutting across the micro, meso, and macro levels [49].

Despite these similarities, there are important differences worth highlighting. First, most of the research capacity-building frameworks are from developed countries, aimed at building research capacity within a given country [2, 46, 50, 51]. This is in contrast to the RSTUVW framework that is informed by research capacity within an N–S context. Therefore, whilst those frameworks can be useful, their applicability in developing countries can be limited due to the unique structural dynamics that characterize N–S collaborations, such as power asymmetry and unethical research conduct [52–55]. Second, those existing frameworks are mainly informed by qualitative interviews [50, 56] and literature reviews [57, 58]. This contrasts with our framework that relies on the real-world experience of the implementers of the UZ-UB collaborative partnership [13]. Third, existing frameworks are mainly focused on research capacity to improve service delivery either at the community level [47, 56] or within health facilities [51, 57]. Whilst these aspects are pertinent in developed and developing countries alike, we have proposed the use of the RSTUVW framework to guide broader aspects that are of particular concern to developing countries, such as health systems strengthening (HSS) [59]. The section below presents the potential wider use of the framework in detail.

### Potential wider use of the RSTUVW framework

The RSTUVW framework has the potential for use in a variety of settings. We propose the potential areas where the framework could be used, as shown in Table 1.

**Table 1:** Potential use of the RSTUVW framework for N–S collaborations and health systems interventions

Area	Room	Skills	Tools	Understanding	Voice	Will
Research capacity-building	**- **Availability of physical space to conduct research --Suitability of the space according to applicable standards - Availability of potential space from strategic partners	**- **Qualified personnel to conduct the research - Portfolio of skills addresses priority policy needs - Skills attained can be transferred to other professionals - Existence of a mentorship programme between the North and South collaborators -Facilitate reverse mentorship where the collaborators from the South transfer local knowledge to their counterparts from the North	**- **Availability of relevant equipment and supplies for research - Availability of reference material and guidelines to conduct the research - Exchange of relevant tools between the collaborators	**- **Availability of personnel that fully understand the rationale for the research - Awareness amongst researchers of their role in shaping policy - An understanding of how the research mentorship fulfils personal career goals - An understanding of the local priorities amongst the collaborators from the North	- Presence of individuals who can champion the programme - Presence of individuals who can articulate the programme in a way that resonates with multiple stakeholders - Presence of individuals with the know-how to navigate the local political and technical context - Presence of parallel individual champions from the North to advocate for the sustenance of the collaboration	- Willingness at technical and policy level to implement the programme - Budgetary allocation and other enabling support for the programme - Alignment of the capacity-building programme with broader developmental priorities - Framing of the capacity-building programme within the context of addressing a pressing societal problem - Alignment of N-S interests at the institutional level to secure long-term will that does not solely rely on individuals
Health systems strengthening	**- **Availability of space to support the crosscutting needs for key health system areas (e.g. health systems delivery, medicines and other health products) - Capacity to modify (e.g. expand, upgrade) relevant space to align with evolving health system needs	- Capacity to diagnose health system problems and put in place a course of action to strengthen the health system - Capacity for strategic thinking, operations management, resource management (financial, physical, material, intellectual) - Capacity for stakeholder engagement and intersectoral collaboration - Capacity to monitor and evaluate progress towards HSS - Capacity to diagnose gaps in HSS efforts and put in place corrective measures	**- **Access to relevant policies and guidelines for HSS **- **Capacity to translate policy and guidelines into locally adapted action plan to respond to own priorities - Capacity to generate local data and document experience to inform policies and guidelines	- Capacity to articulate the rationale for HSS - An understanding of a health system as a complex adaptive system that requires a holistic approach in addressing problems - An understanding of the political dimensions of HSS - Political awareness to anticipate contextual factors that affect HSS efforts - An understanding of the political feasibility of proposed HSS efforts	- Availability of individuals who can champion HSS - Capacity to articulate HSS in a manner that resonates with local and national leaders	- Political will (as demonstrated by the provision of financial, technical and human resources) to support HSS efforts - Willingness of implementers to cope with the challenges of the reform process (e.g. resistance to change amongst other stakeholders, lack of resources, slow progress, fluctuating interest from leaders)
Feasibility of health system intervention	- Availability of space to carry out the intervention - The space can be used for an intervention for a predefined period	- Personnel with skills to implement the intervention - Existence of a programme to refresh the skills to align with evolving priorities - Potential use of the skills to be adapted to other health system area	**- **Availability of equipment and supplies to conduct the programme - Existence of communication technologies to carry out the programme - Existence of communication tools that are compatible with the needs or circumstance of the stakeholders	- An understanding of how the intervention contributes to the broader health goals - An understanding of how the intervention addresses immediate problems - An understanding of the resources required to implement the intervention	- Presence of individuals who can champion the intervention **- **Presence of an individual with recognized credentials to lead the programme **- **The ability to frame the intervention within the context of addressing a pressing health system problem	- Willingness to implement the programme amongst key stakeholders - Budgetary allocation and other enabling support for the programme - Alignment of the intervention with the priorities of key stakeholders
Evaluation of health system intervention	- Was space available to carry out the intervention? - Was there space provided for the intervention for a predefined period?	- Were there personnel with skills to implement the intervention? - Was there existence of a programme to refresh the skills to align with evolving priorities? - Was there an opportunity to use the skills to other health system areas?	**- **Was there availability of equipment and supplies to conduct the programme? - Was there existence of communication technologies to carry out the programme? - Was there existence of communication tools compatible with the needs or circumstances of the stakeholders?	- Was there an understanding of how the intervention contributes to the broader health goals? - Was there an understanding of how the intervention addresses priority problems? - Was there an understanding of the resources required to implement the intervention?	- Were there individuals who championed the intervention? **- **Was there an individual with recognized credentials to lead the intervention? - Was the intervention framed within the context of addressing a pressing health system problem?	- Was there willingness to implement the programme amongst key stakeholders? - Was there a budgetary allocation and other enabling support for the programme? - Was the intervention aligned with the priorities of key stakeholders?

*HSS* health systems strengthening, *N–S* North–South

#### Research capacity-building

The elements of the RSTUVW framework—room, skills, tools, understanding, voice and will—described earlier are therefore applicable to N–S collaborations and do not need further elaboration. However, certain aspects need to be highlighted. First, it is important to emphasize that for N–S collaborations to be effective, there should be deliberate effort to deconstruct the power dynamics that normally characterize such partnerships, where Northern collaborators are often assumed to have a reservoir of knowledge that needs to be transferred to the Southern counterparts. It is therefore critical to establish platforms for reverse mentorship, where knowledge and skills are assumed to be fluid, with either side of the collaborative partnerships poised to benefit from cross-learning. Second, capacity-building is inherently a long-term commitment which relies on technical and policy stability. Stability is therefore required for the champions who provide direct scientific oversight to the programme. This demands the establishment of retention strategies such as flexible tenure for senior figures and career mobility opportunities for young researchers. Stability in the bureaucracy is also important to ensure policy consistency and continuity of the programmes, and where there is instability, N–S collaboration champions should constantly monitor the changes within the policy environment and strategically position their voices to maintain interest in the programmes.

#### HSS

The potential use of RSTUVW within health system environments mimics the N–S settings, but with prospective and retrospective applications. First, the framework can be used prospectively as an assessment tool to gauge the preparedness of the health system or the feasibility of implementing an intervention. The elements of the framework can therefore be used to systematically develop a checklist that can be administered before a programme is implemented, through a desk review, observation or qualitative interview with prospective implementers. After the checklist has been administered, facilitators and enablers of implementation can be mapped out and a feasibility matrix can be developed. The bottlenecks can be categorized according to severity and implication for implementation, which can inform a differential approach to resolve those gaps. For example, some would need to be addressed before the programme starts, whilst others could be addressed during implementation.

#### Feasibility of health system intervention

Second, the framework can be used as a retrospective evaluative tool to check how the programme has performed compared with the original design and pinpoint areas of suboptimal performance. From such analysis, the most important constraints can be isolated and action plans drawn to correct the interventions, including drawing the lessons learned to inform future programmes.

#### Evaluation of health system intervention

Of all the areas, the use of RSTUVW for HSS interventions is arguably the most challenging. This is because HSS interventions involve reform aspects and therefore inherently involve the redistribution of resources—material, physical or intellectual—and are therefore vulnerable to intense competition of interest amongst stakeholders or the “politics” of reform. Thus, whilst N–S collaborations and health system assessments mainly rely on the tangible aspects of the framework, HSS interventions mainly rely on the intangible aspects of the framework to navigate the political landscape for reform. Therefore, use of the framework for HSS interventions should not rely on the importance of the interventions or the evidence to support such interventions. Instead, the framework can be used as a prospective tool at baseline as described above. However, the tangible gaps need to be presented in a way that resonates with the stakeholders that have control over the deployment of resources needed to execute the HSS interventions. Thus, HSS champions need to “think and work politically” [60] to effect the reforms, including lobbying tactics such as strategic framing of problems and establishment of coalitions to jointly advocate for reforms. This requires skills to map who has power, where they get it from and how they are likely to use it to support an identified HSS intervention. Despite the conceptual challenges associated with the use of RSTUVW for HSS, its potential adaptation for the area can be useful, particularly in guiding approaches towards emerging priorities that rely on stronger health systems in the context of developing countries, such as the control of noncommunicable diseases [61, 62].

### Implications of our study

This study has several policy implications. First, it demonstrates that sustainable N–S research collaborations rely on individuals with the attributes, skills and strategies of policy entrepreneurs [9]. Much like commercial entrepreneurs, these are actors who have an inherent willingness to invest their resources—time, energy, reputation—in the hope of a future return. Second, the study underscores the need for a long-term horizon in planning and implementing N–S collaborations. As we have highlighted, the UZ-UB collaboration has spanned over 20 years. The lessons learned for sustaining that long-term interest include alignment of collaborative partnerships with long-term developmental aspiration in Zimbabwe. The UZ-UB partnership was framed as addressing the HIV/AIDS epidemic crisis—a societal concern—instead of a research–focused pharmacotherapy initiative. Third, our study demonstrates that collaborative research partnerships need to fit into national health system frameworks, with involvement of collaborators at various levels of decision-making processes to ensure that their voice is always represented. Whilst these implications are drawn from an in-depth single case study of Zimbabwe, the experience has potential to inform N–S research collaborations in other developing countries and in other areas outside HIV/AIDS pharmacotherapy.

### Limitations of the study and areas for further research

We have presented a novel framework that can be used to assess the sustainability of capacity-building programmes for health research in low-resource settings based on our experience in Zimbabwe. However, some limitations need to be acknowledged. First, the framework is based on the experience for a programme that was initiated when HIV/AIDS was considered a national crisis and political problem, which could explain the support at launch and the subsequent interest over time. The applicability of the framework in noncrisis situations and less politically sensitive programmes remains unknown, and requires further research. Second, in terms of applicability, the framework may fall short of addressing complex policy problems, particularly those that are structurally driven, such as health inequities, or those where macro-level redistributive policies are necessary such as universal health coverage (UHC). Despite those potential limitations, the framework can strategically supplement frameworks that seek an in-depth understanding of policy dynamics, such as political economy models. Third, the RSTUVW framework was informed by a programme that involved long-term funding from external sources. The applicability of the framework within domestically funded programmes remains unclear.

## Conclusion

We leveraged our 20-year experience at implementing the UZ-UB capacity-building programme to propose a novel RSTUVW framework that helps to guide N–S capacity-building for health research. The availability of dedicated requisite physical space (room), development of clearly defined skills, and provision of the necessary tools through technology transfer are all critical elements that ensure effective, impactful and sustainable capacity-building. Key driving values include a clear understanding of the programme goals and a compelling vision; attracting the buy-in and involvement of the most influential voices; and mobilization of a coalition of willing self-driven actors. Dedicated policy entrepreneurs who tenaciously commit their intellectual and material resources to drive change are central in driving the values. We therefore define RSTUVW as a mnemonic-based framework that is useful for systematic framing and guidance in the implementation of an idea, a project proposal, an initiative or a programme at the formative or summative evaluation stages within a research and health system environment.

## Data Availability

Data analysed during this study are included in this published article and its Additional files.

## Abbreviations

AITRP: AIDS International Training and Research Program
ART: Antiretroviral therapy
COHRED: Council on Health Research for Development
GFHR: Global Forum on Health Research
HRTP: HIV Research Training Program
HSS: Health systems strengthening
MCAZ: Medicines Control Authority of Zimbabwe
MOU: Memorandum of understanding
NIH: National Institutes of Health
NIAID: National Institute of Allergy and Infectious Diseases
N–S: North–South
RSTUVW: Room, skills, tools, understanding, voice, will
SDGs: Sustainable Development Goals
SUNY: State University of New York
UB: State University of New York at Buffalo
UHC: Universal health coverage
UZ: University of Zimbabwe
